# Ketamine for bipolar depression: an updated systematic review

**DOI:** 10.1177/20451253231202723

**Published:** 2023-09-26

**Authors:** Farhan Fancy, Sipan Haikazian, Danica E. Johnson, David C. J. Chen-Li, Anastasia Levinta, Muhammad I. Husain, Rodrigo B. Mansur, Joshua D. Rosenblat

**Affiliations:** Mood Disorders Psychopharmacology Unit, University Health Network, Toronto, ON, Canada; Institute of Medical Science, University of Toronto, Toronto, ON, Canada; Mood Disorders Psychopharmacology Unit, University Health Network, Toronto, ON, Canada; Institute of Medical Science, University of Toronto, Toronto, ON, Canada; Mood Disorders Psychopharmacology Unit, University Health Network, Toronto, ON, Canada; Institute of Medical Science, University of Toronto, Toronto, ON, Canada; Mood Disorders Psychopharmacology Unit, University Health Network, Toronto, ON, Canada; Institute of Medical Science, University of Toronto, Toronto, ON, Canada; Mood Disorders Psychopharmacology Unit, University Health Network, Toronto, ON, Canada; Department of Psychiatry, University of Toronto, Toronto, ON, Canada; Institute of Medical Science, University of Toronto, Toronto, ON, Canada; Department of Psychiatry, University of Toronto, Toronto, ON, Canada; Department of Pharmacology and Toxicology, University of Toronto, Toronto, ON, Canada; Campbell Family Mental Health Research Institute, Centre for Addiction and Mental Health, Toronto, ON, Canada; Mood Disorders Psychopharmacology Unit, University Health Network, Toronto, ON, Canada; Institute of Medical Science, University of Toronto, Toronto, ON, Canada; Department of Psychiatry, University of Toronto, Toronto, ON, Canada; Department of Psychiatry, University of Toronto, Toronto, ON, Canada; Mood Disorders Psychopharmacology Unit, Poul Hansen Family Centre for Depression, University Health Network, 399 Bathurst Street, Toronto, ON M5T 2S8, Canada; Department of Pharmacology and Toxicology, University of Toronto, Toronto, ON, Canada; Braxia Scientific, Braxia Health, Canadian Rapid Treatment Centre of Excellence, Mississauga, ON, Canada

**Keywords:** bipolar depression, depression, ketamine, suicide, systematic review

## Abstract

**Background:**

The therapeutic potential of subanesthetic doses of ketamine appears promising in unipolar depression; however, its effectiveness in treating bipolar depression (BD) remains uncertain.

**Objective:**

This systematic review aimed to summarize findings on the use of ketamine for the treatment of BD by assessing its efficacy, safety, and tolerability.

**Design:**

Systematic review.

**Methods:**

We conducted a systematic review of studies that investigated the use of ketamine for adults with BD. We searched PubMed and Embase for relevant randomized-controlled trials, open-label trials, and retrospective chart analyses published from inception to 13 March 2023.

**Results:**

Eight studies were identified [pooled *n* = 235; mean (SD) age: 45.55 (5.54)]. All participants who received intravenous (IV) ketamine were administered a dose of 0.5–0.75 mg/kg as an adjunctive treatment to a mood-stabilizing agent, whereas participants who received esketamine were administered a dosage ranging from 28 to 84 mg. Flexible dosing was used in real-world analyses. A total of 48% of participants receiving ketamine achieved a response (defined as ⩾50% reduction in baseline depression severity), whereas only 5% achieved a response with a placebo. Real-world studies demonstrated lower rates of response (30%) compared to the average across clinical trials (63%). Reductions in suicidal ideation were noted in some studies, although not all findings were statistically significant. Ketamine and esketamine were well tolerated in most participants; however, six participants (2% of the overall sample pool, 5 receiving ketamine) developed hypomanic/manic symptoms after infusions. Significant dissociative symptoms were observed at the 40-min mark in some trials.

**Conclusion:**

Preliminary evidence suggests IV ketamine as being safe and effective for the treatment of BD. Future studies should focus on investigating the effects of repeated acute and maintenance infusions using a randomized study design.

## Introduction

Bipolar depression (BD) is an enduring and debilitating mental illness, a leading cause of global disability, affecting 2–3% of the world population.^1–3^ Nevertheless, over one-third of patients fail to adequately respond to multiple first-line treatment options and are subsequently categorized as having treatment-resistant bipolar depression (TRBD).^2,4–6^ While there is a clear consensus for the definition of treatment-resistant depression (TRD), the same does not apply to the definition of TRBD, for which there are various consensus definitions.^7^ Although BD is of significant concern, there are currently only five Food and Drug Administration (FDA)-approved treatment options available.^8^ Although some trials have suggested the effectiveness of electroconvulsive therapy and repetitive transcranial magnetic stimulation for BD,^9,10^ more research is needed to arrive at conclusive results.

Despite lithium’s effectiveness in lowering suicide rates for BD, patients continue to face elevated suicide risks (23–26% attempting suicide and 5–10% completing suicide).^4,11–13^ While pharmacotherapy (e.g. conventional mood stabilizers and antipsychotics) is the primary treatment option for BD, there are significant challenges and limitations such as treatment resistance, relapse, and several adverse side effects including metabolic issues and cognitive impairments.^2,4,14^ Therefore, there is a pressing demand for novel therapeutic options for BD with a focus on interventions offering improved antidepressant and anti-suicidal efficacy while minimizing adverse side effects and consideration for treatment-emergent mania.^4,8,15,16^

Ketamine is a dissociative anesthetic originally developed as an anesthetic agent; however, recent evidence additionally highlights its rapid-acting antidepressant effects at sub-anesthetic doses (e.g. 0.5–1.0 mg/kg infused over 40–60 min).^8,17,18^ Ketamine’s mechanism of action primarily involves modulation of the glutamatergic system, rather than the monoamine systems typically targeted by conventional antidepressants.^19,20^ Moreover, its mechanism of action goes beyond simple *N*-methyl-D-aspartate antagonism itself, involving multiple other mechanisms (involving brain-derived neurotrophic factor, glycogen synthase kinase-3 beta, and membrane ionic influx).^21^

Over the past two decades, there has been a resurgence of research supporting the use of sub-anesthetic doses of intravenous (IV) ketamine for major depressive disorder (MDD) and TRD.^22–24^ However, the majority of randomized controlled trials (RCTs) examining ketamine have been limited to single-dose proof-of-concept studies looking at the adult TRD population.^4^ More recently, the importance of a repeated doses regimen (twice or thrice weekly over 2 weeks) has shown to produce more pronounced and sustained antidepressant effects – approximately doubling the antidepressant response rate.^4,25–28^

As outlined in recent reviews, RCT findings primarily support the effectiveness of IV ketamine in MDD, but it remains unclear whether the same holds true for BD.^1,23,29^ Furthermore, the generalizability of these findings in the real-world setting remains uncertain given the increased complexity and comorbidity observed in the TRBD population.^4^ Despite previous reviews examining the potential of ketamine as a treatment for TRBD,^30^ recent advancements in ketamine research necessitate an updated evaluation of its therapeutic value through RCTs and real-world effectiveness (RWE) data. The importance of RWE data has been increasingly emphasized and recognized as an essential companion to RCTs, as it provides a more comprehensive understanding of the practical implications of using ketamine for BD.^31^

For the current invited review, our goal is to present an updated summary of the research on the efficacy and safety of ketamine in treating BD. A systematic review that synthesizes findings from RCTs, open-label studies, and RWE data will be conducted.

## Methods

### Protocol and registration

This study was recorded on the Open Science Framework (https://osf.io/ksvnb/) and adhered to Preferred Reporting Items for Systematic Reviews and Meta-analyses guidelines for systematic reviews.^32^

### Eligibility criteria

Our inclusion criterion was restricted to studies that investigated the use of ketamine in any form in adults (18 years and older) for the treatment of BD. RCTs, open-label trials, retrospective chart analyses, and RWE studies that evaluated the use of ketamine as a stand-alone treatment or in conjunction with psychotherapy were considered. Research designs involving surveys, cohort studies, case series, reviews, post hoc/secondary analyses, commentaries, and clinical overviews were excluded. In addition, studies needed to report at least one outcome related to the efficacy or safety of ketamine (e.g. response to treatment or adverse events). Finally, studies failing to differentiate between unipolar and BD were also excluded.

### Information sources and search

To gather relevant studies for our research, we conducted a search of multiple databases, including PubMed and Embase spanning from the inception of these databases to 13 March 2023, using the following medical search heading (MeSH) terms and search strings: [[[(ketamine) OR (esketamine)] OR (arketamine)] OR (racemic ketamine)] AND (bipolar depression). To identify any ongoing or unpublished studies, we also searched ClinicalTrials.gov, the EU Clinical Trials Register, and the Australian and New Zealand Clinical Trials Registry using the keywords ‘ketamine’ and ‘bipolar depression’. In addition, we manually searched the reference lists of the included studies and relevant reviews to find potentially applicable articles.

### Study selection

Using the web-based systematic review tool Covidence (Veritas Health Innovation), two researchers (FF and SH) individually reviewed titles and abstracts to identify relevant articles. The co-authors independently evaluated the full texts of the selected articles for inclusion. In the event of any discrepancies, they engaged in a discussion to reconcile their differences and come to a consensus.

### Data collection process and data items

Using a custom data extraction template in Microsoft Excel that captured patient demographics, response and remission rates, and adverse events, three co-authors (FF, SH, and DC) gathered information on various aspects of the studies including interventions, study outcomes, ketamine dosage, study withdrawals, and adverse events.

### Risk of bias in individual studies

To evaluate the potential for bias within each trial, we utilized the Cochrane risk of bias tool for RCTs. This tool evaluates various factors, including selection bias, performance bias, detection bias, attrition bias, and reporting bias.^33^ Two authors (DC and DJ) independently conducted the risk of bias assessments. In case of any discrepancies between the two authors, they discussed to come to a consensus.

## Results

### Study selection

**Figure 1.**
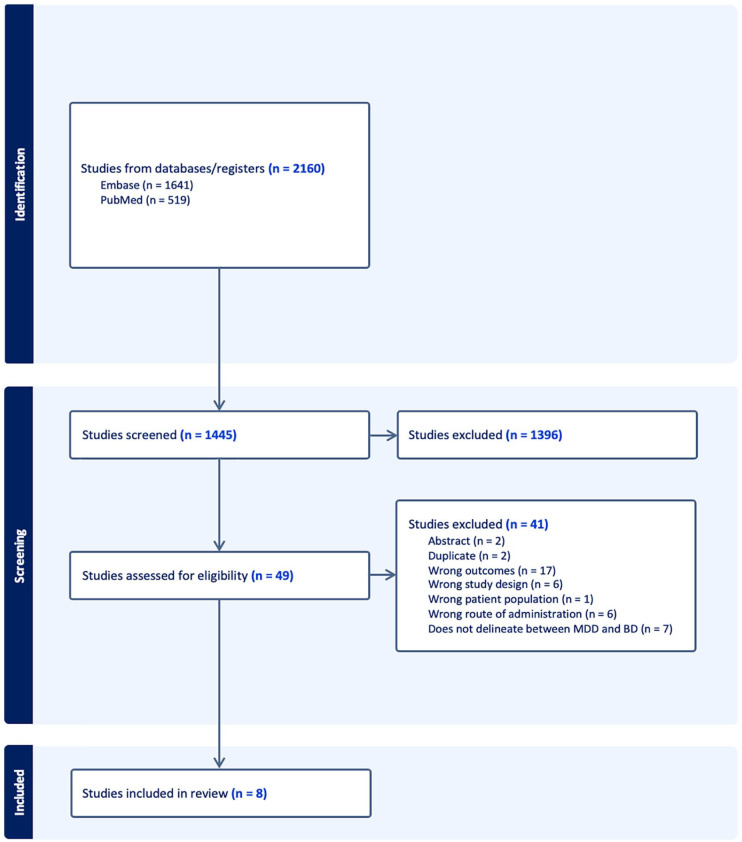
Preferred Reporting Items for Systematic Reviews and Meta-analyses flow diagram outlining the systematic review.

Using the outlined search strategy, a total of 2160 records were identified (Figure 1). After duplicates were removed, 1445 records remained. A title and abstract screening was done on these records, leaving 47 records for a full-text review. Eight studies met the inclusion criteria and were ultimately included in our analysis.

### Characteristics of studies, participants, and interventions

Table 1 shows the characteristics of the studies included in this analysis. Three studies were RCTs,^24,34,35^ three were open-label, single-arm,^36–38^ one study was an open-label, double-arm, real-world observational trial,^39^ and one was a real-world observational trial.^4^ Four studies^34,35,38,40^ included single ketamine infusions, whereas three studies^4,36,37^ included multiple infusions. Seven studies^4,34–38,40^ used racemic intravenous ketamine as the intervention, whereas one study used an esketamine nasal spray.^39^

**Table 1.** Characteristics of studies included.

Study	Design	Population	Intervention(s)	Primary findings
Diazgranados *et al.*, 2010	Crossover RCT	TRBD (*n* = 17)	Racemic, adjunctive ketamine: 0.5 mg/kg IV on two test days 2 weeks apart *versus* placebo	71% responded to ketamine at some point during the trial. One participant in each group developed manic symptoms, and some participants experienced dissociative symptoms at the 40-min mark
Zarate *et al.*, 2012	Crossover RCT	TRBD (*n* = 15)	Racemic, adjunctive ketamine: 0.5 mg/kg IV on two test days 2 weeks apart *versus* placebo	79% (*versus* 0%) responded to ketamine (*versus* placebo) during the trial on the MADRS. Dissociative symptoms occurred at the 40-min mark
Grunebaum *et al.*, 2017	Parallel RCT	BD (*n* = 16)	Racemic, adjunctive ketamine infusions dosed at: 0.5 mg/kg IV *versus* midazolam infusions dosed at 0.02 mg/kg	57% demonstrated a response on the Scale for Suicidal Ideation. Depression ratings decreased across the ketamine arm, but the between-group difference was not significant
Rybakowski *et al.*, 2017	Open-label, single-arm trial	TRBD (*n* = 53)	Racemic, adjunctive ketamine: 0.5 mg/kg IV, single dose	24.5% responded to ketamine at 24 h post-infusion, and 51% responded to ketamine at day 7. No significant adverse events were reported
Zheng *et al.*, 2020	Open-label, single-arm trial	TRBD (*n* = 19)	Six racemic, adjunctive ketamine infusions dosed at 0.5 mg/kg. 6 infusions over 12 days	Rates of response and remission were 73.7% and 63.2% at the study end. There were no significant dissociative and psychotomimetic symptoms on the CADSS or BPRS
Wilkowska *et al.*, 2021	Open-label naturalistic observational study	TRBD (*n* = 13)	Eight racemic, adjunctive ketamine infusions: 0.5 mg/kg twice a week over 4 weeks	Following the seventh infusion, 61.5% of participants responded. No serious adverse events were observed
Martinotti *et al.*, 2023	Open-label double-arm observational trial	TRBD (*n* = 35) *versus* TRD (*n* = 35)	Two doses of intranasal esketamine were administered per week in the first month, and one dose per week in the following 2 months (28–84 mg)	A significant reduction in depressive symptoms was found at 1 month and at 3 months compared to baseline, with no significant differences in response or remission rates between subjects with TRBD and TRD. Esketamine showed a greater anxiolytic action in subjects with TRBD than in those with TRD. The low risk of manic switch in BTRD patients confirmed the safety of this treatment
Fancy *et al.*, 2023	Open-label single-arm observational trial	TRBD (*n* = 66)	Two infusions of ketamine hydrochloride 0.5 mg/kg diluted in 0.9% saline solution infused over 40 min, with a potential to increase the dose up to 0.75 mg/kg for the third and fourth infusions if inadequate response	Response rate (QIDS-SR16 total score decrease ⩾50% from baseline) was 35% and remission rate (QIDS-SR16 total score ⩽5) was 20% after four infusions. Infusions were generally well tolerated with treatment-emergent hypomania observed in only three patients (4.5%) with zero cases of mania or psychosis

BPRS, Brief Psychiatric Rating Scale; BTRD, Bipolar Treatment Resistant Depression; CADSS, Clinical-Administered Dissociative Symptoms Scale; MADRS, Montgomery-Åsberg Depression Rating Scale; RCT, randomized controlled trial; TRBD, treatment-resistant bipolar depression; TRD, treatment-resistant depression; QIDS, Quick Inventory of Depressive Symptoms.

A total of 235 participants were included [mean (SD) age: 45.5 (5.53); 60.9% female]. With the exception of one study,^35^ all participants had Diagnostic and Statistical Manual of Mental Disorder IV- or 5-diagnosed BD that failed to respond to one or more complete trials of mood stabilizing medications. Six clinical trial studies used racemic ketamine at a dose of 0.5 mg/kg delivered intravenously with an adjunctive mood stabilizer that was used throughout ketamine treatment.^34–38,40^ In the RWE trial, participants had the potential to receive a maximum dose of 0.75 mg/kg if sufficient response was not achieved after the first two doses.^4^ In the two-arm observational trial^39^ of patients with TRBD and TRD (the latter of which is excluded from this review), participants received a variable number of esketamine doses at a dosage of 28–84 mg (see Table 4 in Martinotti and colleagues’ report). The nasal spray formulation for the Italian esketamine study (ESK-NS) consisted of two doses of intranasal esketamine administered per week in the first month, and one dose per week in the following 2 months (28–84 mg).^39^ All racemic ketamine trials excluded participants who had psychotic symptoms or a history of substance abuse, and most studies excluded those who had comorbid general medical conditions or who were pregnant or breastfeeding. In the esketamine trial,^39^ patients with comorbid physical conditions that are contraindicated to esketamine treatment were excluded (Tables 2 and 3).

**Table 2.** Risk of bias assessment for included RCTs as per Cochrane’s risk of bias tool.

Source	Domain 1: risk of bias from the randomization process	Domain 2: risk of bias due to deviations from the intended interventions	Domain 3: risk of bias due to missing outcome data	Domain 4: risk of bias in the measurement of the outcome	Domain 5: risk of bias in the selection of the reported result
Diazgranados *et al.*, 2010	Low	Low	Moderate	Low	Low
Zarate *et al.*, 2012	Low	Low	Moderate	Low	Low
Grunebaum *et al.*, 2017	Low	Low	Moderate	Low	Low

RCT, randomized controlled trial.

**Table 3.** Risk of bias assessment for included open-label studies as per Cochrane’s risk of bias in non-randomized studies – of interventions tool.

Source	Domain 1: risk of bias due to confounding	Domain 2: risk of bias due to selection of participants	Domain 3: risk of bias due to classification of interventions	Domain 4: risk of bias due to deviations from intended interventions	Domain 5: risk of bias due to missing data	Domain 6: risk of bias due to measurement outcomes	Domain 7: risk of bias due to selection of reported outcomes
Rybakowski *et al.*, 2017	Moderate	Low	Low	Low	Low	Moderate	Low
Zheng *et al.*, 2020	Moderate	Low	Low	Low	Serious	Moderate	Low
Wilkowska *et al.*, 2021	Low	Low	Low	Low	Moderate	Low	Low
Martinotti *et al*., 2023	Low	Low	Low	Low	Low	Low	Low
Fancy *et al.*, 2023	Low	Moderate	Low	Low	Low	Low	Low

**Table 4.** Ongoing clinical trials of ketamine for bipolar depression.

Study	NCT#/EudraCT#	Location(s)	Study drug(s)	Primary outcome	Study design	*N*	Key dates
Repeated Ketamine Infusions for Treatment-Resistant Bipolar Disorder: A Randomized, Double-Blind, Midazolam-Controlled Phase II Clinical Trial (KET-BD)	NCT05004896	Toronto, Canada	Flexibly dosed, twice weekly ketamine infusions (0.5–0.75 mg/kg) over 2 weeks	Change in mean MADRS at day 14 between ketamine and midazolam arm	Clinical: randomized, double-blind, placebo-controlled	100	Start date: April 2022 Primary completion date: April 2024 Study completion date: December 2024
Maintenance Ketamine Infusions for Treatment-Resistant Bipolar Depression: An Open-Label Extension Trial	NCT05339074	Toronto, Canada	Flexibly dosed, bi-weekly ketamine infusions (0.5–1.0 mg/kg) over 3 months	Change in mean MADRS over 12 weeks	Open-label	60	Start date: August 2022 Primary completion date: June 2024 Study completion date: August 2024
Open Study of the Neurobiological Effects of Intranasal Ketamine in Children and Adults with Bipolar Disorder – Fear of Harm Phenotype	NCT05209217	Belmont, MA, USA	Intranasal administration of customary prescribed dose	Bold fMRI response in the amygdala and posterior insula	Observational, perspective, cohort	20	Start date: June 2019 Primary completion date: December 2022 Study completion date: January 2023
Neural Correlates of Ketamine’s Anti-Suicidal Effects in Bipolar Depression (DEEPP)	NCT05177146	Toronto, Canada	IV ketamine administered twice per week for 4 weeks	Impact of IV ketamine on intracortical facilitation	Open-label	30	Start date: May 2022 Primary completion date: February 2024 Study completion date: August 2024
The BIO-K Study: A Single-Arm, Open-Label, Biomarker Development Clinical Trial of Ketamine for Non-Psychotic Unipolar Major Depression and Bipolar I or II Depression (BIO-K)	NCT03156504	Mayo Clinic, USA	IV Ketamine administered three times in 1 week	Total number of subjects who achieved remission after three infusions of ketamine	Open-label	75	Start date: 1 June 2017 Primary completion date: February 2020 Study completion date: March 2020
Central *versus* Peripheral GABA and Glutamate Biomarkers for Treatment Response During Two Infusions of Intravenous Ketamine for Treatment-Resistant Depression	NCT03573349	Mayo Clinic, USA	Two ketamine infusions (0.5 mg/kg)	Percent change in the anterior cingulate cortex GABA and glutamate during a 40 min ketamine infusion	Open-label	20	Start date: January 2019 Primary completion date: Study completion date: December 2023
NRX100 *versus* Placebo for Rapid Stabilization of Acute Suicidal Ideation and behavior in Bipolar Depression	NCT03396601	Hollywood, Fl; Fort Worth, Tx, USA	Single infusion ketamine (0.5 mg/kg) *versus* placebo	Change in suicidal ideation, as measured by the C-SSRS, after ketamine infusion	Clinical: randomized, double-blind, placebo-controlled	150	Start date: June 2019 Primary completion date: March 2022 Study completion date: April 2022
Ketamine as an Adjunctive Therapy for Major Depression	NCT04939649	Dublin, Ireland	4-week course of twice-weekly studies	Change in MADRS over 28 weeks between groups	Clinical: randomized, double-blind, placebo-controlled	104	Start date: September 2021 Primary completion date: October 2023 Study completion date: April 2024
Clinical Predictors of Intravenous Ketamine Response in Treatment-Resistant Depression	NCT05625555	Halifax, Nova Scotia	Single ketamine infusion (0.5 mg/kg) over 40 min *versus* midazolam	Change in MADRS over 2 weeks between groups	Clinical: randomized, double-blind, placebo-controlled	40	Start date: September 2023 Primary completion date: September 2024 Study completion date: December 2024
Naturalistic Study of Ketamine in the Treatment of Depression	NCT05249309	Porto Alegre, Brazil	Twice-weekly doses of ketamine (0.5 mg/kg) administered subcutaneously for 1 month	Change in C-SSRS over 24 weeks	Open-label, naturalistic	90	Start date: May 2021 Primary completion date: November 2022 Study completion date: March 2023
Observation of Ketamine Treatment Safety and Tolerability in Adult Psychiatry Clinic Medical University of Gdańsk Inpatients (KetGD)	NCT05565352	Gdańsk, Poland	IV: ketamine will be infused (slow IV infusions of ketamine (0.5 mg/kg) over 40 min) twice weekly over a period of 4 weeks) Nasal: ketamine will be given in intranasal spray twice weekly over a period of 4 weeks Oral: ketamine will be given orally (solution 2.0 mg/kg, 2.5 mg/kg) twice weekly over a period of 4 weeks	Incidence of adverse events assessed by CADSS (Time Frame: Baseline through week 5)	Open-label naturalistic cohort	140	Start date: September 2022 Primary completion date: December 2027 Study completion date: December 2027
Efficacy and Feasibility of Intranasal Ketamine on Acute Suicidality, a Double Blind Randomized Placebo-Controlled Trial (Ketamine Trial for Acute Suicidality, KETA)	EudraCT number: 2020-002905-24	Groningen, Netherlands	75 mg intranasal ketamine administration compared to 4.0 mg intranasal midazolam (placebo)	Change in suicidality scores on the BSSI between baseline and 180 minutes after 75 mg intranasal ketamine administration compared to 4.0 mg intranasal midazolam (placebo)	Double-blind randomized placebo-controlled trial	112	Start date: October 2021

BSSI, Beck Scale for Suicidal Ideation; CADSS, Clinical-Administered Dissociative Symptoms Scale; C-SSRS, Columbia Suicide Severity Rating Scale; fMRI, functional Magnetic Resonance Imaging; GABA, Gamma-Aminobutyric Acid; IV, intravenous; MADRS, Montgomery-Åsberg Depression Rating Scale.

### Efficacy of IV ketamine in clinical trials

The overall pooled response rate (defined as those having a reduction in their baseline depression severity of at least 50%) across all eight studies was 48% for those receiving ketamine (94/203). The range of response rates across studies varied from 26%^4^ to 79%.^40^ For the three studies involving control groups,^24,34,35^ the overall placebo-pooled response rate was 5% (2/42). The efficacy of single-dose ketamine did not extend beyond the 2-week mark; however, studies administering repeated infusions of ketamine over 2 weeks showed longer-lasting efficacy, up to and including a month after initial administration.^36^

Large reductions were found in suicidal symptoms after one or more infusions across all trials. However, the significance of these findings varied. In an open-label trial, suicidal ideation was significantly reduced at 4 h post-infusion compared to baseline, with sustained reductions across multiple infusions.^36^ In one RCT, the change in suicidal ideation was not significantly different between the ketamine and midazolam groups.^35^ In the ketamine arm, 57% (4/7) of patients responded and 43% (3/7) were in remission at 1-day post-ketamine infusion. Wilkowska *et al.* reported a significant change in the severity of suicidal thoughts across infusions among those who showed antidepressant response to ketamine, but no significant change in those who did not show a response.^37^

### Efficacy of IV ketamine in real-world analyses

Fancy *et al.*’s real-world observational study (*N* = 66) using four IV ketamine doses of 0.5–0.75 mg/kg over a 2-week period achieved a 35% response rate (⩾50% reduction in the QIDS-SR16 score) and 20% of patients met criteria for remission (QIDS-SR16 ⩽ 5).^4^ Moreover, there was a trend toward greater depressive symptoms reduction in the BD-II group compared to BD-I.

Ketamine was also associated with improved anxiety scores and psychosocial functioning.

A significant effect of infusion for anxiolytic symptoms was observed, as measured by the General Anxiety Disorder 7-item (GAD-7) scale, *F*(2, 82.5) = 8.99, *p* < 0.001, Cohen’s *f* = 0.43). Lastly, there was no significant main effect of BD-I *versus* BD-II subgroups on GAD-7 scores [*F*(1, 56.19) = 1.06, *p* > 0.05].

The Sheehan Disability Scale (SDS) was used to assess psychosocial functioning and was broken down into three separate subcategories: SDS-WORK, SDS-SOCIAL, and SDS-FAMILY, representing different cognitive domains. There was no significant main effect of infusion on the SDS-WORK score [*F*(2, 52.1) = 0.61, *p* > 0.05, Cohen’s *f* = 0]. There was also no significant main effect of BD-I *versus* BD-II subgroups on SDS-WORK scores [*F*(1, 45.9) = 0.27, *p* > 0.05]. There was a significant main effect of infusion for the SDS-SOCIAL score [*F*(2, 76) = 10.83, *p* < 0.001, Cohen’s *f* = 0.50], but no significant main effect of BD-I *versus* BD-II subgroups on SDS-SOCIAL scores [*F*(1, 54.7) = 1.06, *p* > 0.05]. Lastly, there was a significant main effect of infusion for the SDS-FAMILY score [*F*(2, 76.5) = 8.73, *p* < 0.001, Cohen’s *f* = 0.44], but again, there was no significant main effect of BD-I *versus* BD-II subgroups on SDS-FAMILY scores [*F*(1, 64.18) = 2.94, *p* > 0.05].

In the real-world study of esketamine,^39^ response and remission rates at 1 month were 25.7% and 17.14%, with those rates increasing to 68.57% and 48.57% at 2 months post-baseline, respectively.

### Safety and tolerability of IV ketamine for BD

Across clinical trials,^24,34,35^ participants tolerated IV ketamine treatment reasonably well. Common adverse events included perceptual and physical changes such as drowsiness, dizziness, blurred vision, nausea, and headaches, which resolved soon after the end of infusions. Transient increases in blood pressure and heart rate were also noted throughout the trials, which resolved after infusions. Some significant adverse events were noted. For example, two participants in the RCTs (one receiving ketamine and one receiving placebo) developed manic symptoms.^34^ One participant (2.85%) in the open-label, two-arm observational study experienced an affective switch.^39^ Participants in two trials also developed significant dissociative symptoms^34,40^; however, this was not noted in other trials. With regard to RWE data, infusions were well tolerated with treatment-emergent hypomania observed in only 3/66 patients (4.5%) with zero cases of mania or psychosis.^4^

## Discussion

### Efficacy

To our knowledge, this systematic review is the latest to investigate the efficacy, safety, and tolerability of IV ketamine in treating BD. Based on the analysis of the eight included studies, our results suggest that IV ketamine is a promising, safe, and effective treatment option for BD and TRBD. The overall pooled response rate across all eight studies was 48% for those receiving ketamine (94/203). The overall range of response rates varied from 35% to 79% across the included studies.

The wide range of the overall response rate across studies can be attributed to several factors. One explanation for the wide range of response rates could be due to inherent variability in the levels of treatment resistance of participants across the different studies.^4^ The lower end of the overall response rate for RWE analysis is conservative and more realistic compared to the earlier studies reflecting the complex BD patient population.^40^ In the RWE analysis, study inclusion criteria were less strict, representing a more ‘real-world’ sample and the study also permitted a dose increase after the first two infusions, providing another avenue for the discrepancies.^4^ In addition, the small sample size of (*n* = 15) in prior trials may be another reason why results varied from the RWE study, which had a sample size of 66 participants. The RWE analysis was the first study delineating between type I and type II BD and to our knowledge, this study represents the largest sample size of the well-characterized group of adults with TRBD receiving repeated ketamine infusions at a community-based clinic.^4^ A key finding in this delineation was a trend toward a higher reduction of depressive symptoms in the BD-II group compared to BD-I; nonetheless, more studies with larger sample sizes are needed to confirm this trend.^4^ Taken together, our results support prior findings indicating that IV ketamine infusions were associated with significant improvements in depressive symptoms in adults with BD.

### Single *versus* repeated doses

There is currently limited data available on repeated administration of IV ketamine for BD. The evidence to date for the use of repeated ketamine infusions for BD is derived primarily from open-label studies with conflicting results.^4^ To this end, future RCT designs taking into consideration both acute and maintenance treatments are needed to further evaluate the long-term benefits and implications.

### Suicidality

Ketamine’s anti-suicidal effects are gaining recognition, especially in the context of BD, a psychiatric illness that is frequently linked with a high risk of suicide.^8,15,41–43^ While reductions in suicidal symptoms after one or more infusions across trials varied in significance, it is important to note the downward trend across all studies. Lastly, no participants reported an increase in their preexisting suicidal thoughts following ketamine infusions.

### Anxiety

In addition to its rapid anti-depressive and anti-suicidal effects, ketamine also leads to improved anxiety symptoms as seen in Fancy and colleague’s RWE analysis.^4^ They observed a significant four-point reduction from baseline to post-treatment assessment visit in GAD-7 scores suggesting a reduction from severe to moderate anxiety.^4^ While there are currently no studies specifically investigating the effects of repeated ketamine infusions on anxiety, our findings are consistent with other trials suggesting the potential of ketamine to alleviate anxiolytic symptoms.^3,6,42,44,45^

### Psychosocial functioning

With respect to psychosocial functioning, a RWE study reported significant improvement in SDS ‘social’ and ‘family’ subdomains, but no improvements in the ‘work’ subdomain.^4^ One explanation for this may be that workplace functioning has a longer latency to improvement due to practical reasons (e.g. individuals needing time to find a job after recovery).^4^ Moreover, ketamine’s anti-suicidal effects are complex and may be related to cognition improvement as well.^45–47^ Overall, there is insufficient reporting in the current literature to draw conclusive findings on ketamine’s effect on psychosocial functioning. Further investigation is warranted to explore the potential psychosocial and interpersonal benefits of ketamine infusions in patients with BD.

### Safety and tolerability

While there is a growing body of research supporting the use of ketamine as an antidepressant for MDD, some researchers have been hesitant to include bipolar-depressed patients in these studies due to concerns about the risk of treatment-emergent affective switching and the risk of dissociation. Despite these concerns, this review demonstrates that recent research supports a relatively low risk of manic switching and dissociation with ketamine treatment. The majority of studies suggest that IV ketamine is a generally well-tolerated treatment for BD.

Of the studies included in this review, only two reported instances of treatment-emergent affective switching. One RCT included in this review reported that one participant receiving ketamine developed manic symptoms,^34^ and a RWE study reported three cases of hypomania after the third or fourth ketamine infusion.^4^ Therefore, of the 235 participants in the eight studies included in this review, only 1.7% developed manic/hypomanic symptoms, which is lower than the 3–10% incidence of manic switching seen with traditional antidepressant medications and placebo.^5,48–50^

It is important to note that while the risk of manic switching with IV ketamine treatment is relatively low, it is still a potential risk that should be closely monitored and further characterized in future trials. Patients with BD who receive ketamine should be assessed post-infusion for signs of mood elevation with a well-validated tool for assessing mania, such as the Young Mania Rating Scale.

Only two trials reported participants developing significant dissociative symptoms, which typically occurred 40 min after receiving the ketamine infusion.^34,40^ Nevertheless, in the remaining four trials, there were no significant observations of dissociation or manic switch throughout the duration of the studies. While standard antidepressants can induce rapid cycling, it is uncertain whether ketamine can induce the same, as trials involving ketamine are usually brief. While there have been instances of manic switching in some participants who received a single ketamine infusion, the small sample sizes in these cases may be insufficiently powered to identify manic switching. However, one study involving 98 participants with MDD found insufficient evidence to support the induction of mania through a single sub-anesthetic dose of ketamine.^51^

Despite the potential risk of manic switch and dissociation, adults with TRBD should be included in studies assessing the antidepressant effect of ketamine. BD is a debilitating condition that affects a significant portion of the population, and approximately one-third of patients will not experience syndromal relief with multiple first-line therapies, resulting in a diagnosis of TRBD. Thus, it is essential to investigate potential treatment options for patients who do not respond to standard treatments.

### Limitations

Although there are some strengths to this review, it is important to note several fundamental limitations. First, publication bias may have influenced our findings, as studies showing no or minimal improvements from IV ketamine may have been overlooked. Second, although we made an effort to cover a significant follow-up period after ketamine treatment administration, we were unable to obtain comprehensive information beyond the 2-week mark. Therefore, our review’s results are constrained to this timeframe. Third, the participants from which we drew data may not be representative of the broader population with BD because of strict inclusion/exclusion criteria. However, we included an RWE analysis to account for this. While Fancy and colleagues’ RWE analysis^4^ was the first to delineate between type I and type II BD, more RWE research is needed to complement RCT findings to achieve a more holistic understanding of ketamine’s effectiveness. Fourth, it is worth noting that the significant heterogeneity within the included studies may have influenced our results. For instance, there are distinctions between patients with TRBD and those without, as well as differences between those who were treated as inpatients *versus* those treated at community clinics. Fifth, variations exist between studies in which participants received either a single ketamine dose or multiple doses. Sixth, seven of the eight studies used racemic ketamine while one study used the S-enantiomer of ketamine (ESK-NS). Data on the use of arketamine (R-enantiomer of ketamine) remain scarce with there being only one pilot trial to date testing its feasibility and safety for BD.^52^ Thus, there are literature limitations on the formulations of ketamine and its enantiomers and their respective impact on the safety and tolerability of observed outcomes. Lastly, the long-term efficacy, safety, and tolerability of maintenance ketamine infusions have not been fully studied. While promising preliminary evidence exists for the acute antidepressant effects of ketamine for BD, more research is needed to understand its long-term effects.

### Future directions

It is an exciting time for ketamine research in depression, and the literature on ketamine in BD is catching up to the literature on MDD. RCTs evaluating the efficacy of repeated ketamine infusions are needed to determine whether the initial depressive benefits can be sustained, as single-dose ketamine seems to provide transient benefits. Similarly, the effects of maintenance ketamine infusions for long-term antidepressant benefits are required. In addition, the effects of combining ketamine with other non-pharmacotherapeutic treatment options should be explored. Table 4 displays current registered clinical trials investigating the use of ketamine for BD.

## Conclusion

Ketamine is an experimental, innovative treatment for BD with rapid action. This systematic review supports the use of IV racemic ketamine for individuals with BD, based on preliminary evidence. Although several clinical studies have demonstrated significant short-term benefits, the long-term benefits remain inadequately explored. Although ketamine is not currently approved by the FDA for any mental disorder, its isomer, esketamine, is the first FDA-approved non-monoamine-based psychotropic agent for adults with TRD. Nevertheless, ketamine is recommended for MDD as a treatment option when two prior antidepressants are insufficient (i.e. treatment-resistant). More RCTs, as well as RWE data, are necessary to explore the efficacy and safety of administering IV ketamine for treating BD.

## Declarations

**Ethics approval and consent to participate:** Not applicable.

**Consent for publication:** Not applicable.

**Competing interests:** The authors declared the following potential conflicts of interest with respect to the research, authorship, and/or publication of this article: Dr. JDR has received research grant support from the Canadian Institute of Health Research (CIHR), Physician Services Inc (PSI) Foundation, Labatt Brain Health Network, Brain and Cognition Discovery Foundation (BCDF), Canadian Cancer Society, Canadian Psychiatric Association, Academic Scholars Award, American Psychiatric Association, American Society of Psychopharmacology, University of Toronto, University Health Network Centre for Mental Health, Joseph M. West Family Memorial Fund and Timeposters Fellowship and industry funding for speaker/consultation/research fees from Janssen, Allergan, Lundbeck, Sunovion, and COMPASS. He is the Chief Medical and Scientific Officer of Braxia Scientific and the medical director of the Canadian Rapid Treatment Centre of Excellence (Braxia Health). Dr. MIH receives research support from the Brain and Behavior Research Foundation, Canadian Institutes of Health Research (CIHR), CAMH Foundation, Grand Challenges Canada, the PSI Foundation, and the University of Toronto. He has conducted contracted research for COMPASS Pathfinder Limited and provided consultancy to Mindset Pharma, PsychEd Therapeutics, and Wake Network.

**Availability of data and materials:** All data collected is presented in the main manuscript.
